# Purification and Identification of Antioxidant Alcalase-Derived Peptides from Sheep Plasma Proteins

**DOI:** 10.3390/antiox8120592

**Published:** 2019-11-27

**Authors:** Chengli Hou, Liguo Wu, Zhenyu Wang, Elena Saguer, Dequan Zhang

**Affiliations:** 1 Institute of Food Science and Technology, Chinese Academy of Agricultural Sciences, Key Laboratory of Agro-Product Processing, Ministry of Agriculture and Rural Affairs, Beijing 100193, China; houchengli@163.com (C.H.); liguowu911@163.com (L.W.); food2006wzy@163.com (Z.W.); 2 Institut de Tecnologia Agroalimentària (INTEA), Universitat de Girona, C/Maria Aurèlia Capmany 61, 17003 Girona, Spain

**Keywords:** sheep plasma protein, antioxidative peptides, alcalase, purification, mass spectrometry

## Abstract

In this study, sheep plasma was submitted to Alcalase-hydrolysis and peptides with better antioxidant properties measured through both the ferric-reducing antioxidant power (FRAP) and the 2,2-diphenyl-1-picrylhydrazyl (DPPH) radical scavenging ability assays were isolated and identified. After hydrolysate ultrafiltration and semi-preparative reverse-phase high-performance liquid chromatography, nine fractions (F1–F9) were obtained, with the two first (F1 and F2) showing the greatest antioxidant potential. These two fractions were further separated by the AKTA purifier system to generate four (F1-1–F1-4) and five (F2-1–F2-5) fractions, respectively, with two of them (F1-2 and F2-1) exhibiting appreciable FRAP activity and DPPH radical scavenging ability. Using liquid chromatography-tandem mass spectrometry, three antioxidant peptides were identified. From their amino acid sequences (QTALVELLK, SLHTLFGDELCK, and MPCTEDYLSLILNR), which include amino acids that have been previously reported as key contributors to the peptide antioxidant properties, it can be maintained that they come mainly from serum albumin. These results suggested that the sheep plasma protein can be considered as a good source of antioxidant peptides and bring forth new possibilities for the utilization of animal blood by-products.

## 1. Introduction

In China, mutton is increasingly consumed due to the improvement of people’s living standard [1]. In 2017, the number of sheep and goats slaughtered has exceeded 300 million, with a mutton production of 4.71 million tons, which supposes increases of 22% and 121% relative to the production in 2007 and 1997, respectively [2]. Sheep blood is one of the main by-products of the mutton industry, which accounts for about 4% of the sheep’s live weight [3]. At present, the utilization rate of sheep blood is very low. It is mainly used in the manufacture of traditional foods like blood sausages, blood tofu, blood rake or for the processing of animal feed [4,5], while most of the sheep’s blood is discarded directly, causing great waste of resources and environmental pollution [6].

Some blood plasma characteristics, particularly its richness in proteins of high biological value with good techno-functional properties and/or interesting biological activities, justify the interest for plasma valorization, besides being advantageous from an economic point of view [3]. In this sense, previous studies have actually shown that plasma is an important source of bioactive peptides with antioxidant, antibacterial, antihypertensive, and other biological properties after enzymatic hydrolysis using exogenous proteases [7,8], without entailing relevant changes at a nutritional level [9,10]. Bah et al. [3] found that deer and porcine plasma protein hydrolysates obtained after the enzymatic treatment with plant and fungal proteases are strong antioxidants. Sheep plasma protein hydrolysates (SPPH) also have antioxidant properties, with the Alcalase-produced ones showing significantly higher antioxidant activity than those ones obtained with neutral and acidic proteases [11]. Alcalase (also known as subtilisin A) is a serine endopeptidase from *Bacillus licheniformis* with an alkaline optimum pH and a broad substrate specificity, that has been shown useful to obtain peptides with antioxidant activity from different protein sources [12,13,14,15,16]. Interestingly, Alcalase-derived SPPH was able to inhibit both lipid and protein oxidation and, this way, to improve the color stability in mutton patties [17].

These results would be consistent with the amino acid sequences of the main sheep plasma proteins (serum albumin, globulins, and fibrinogen) released in the National Centre for Biotechnology Information (NCBI) database and with the reported amino acid sequences of many antioxidant peptides [18]. In this sense, it is important to note that the composition and the amino acid sequence are amongst the most relevant factors influencing peptide bioactivity [19,20,21]. Peptides with a high proportion of hydrophobic amino acids frequently exhibit high antioxidant activity [21]. However, both acidic (Glu) and basic (Arg) amino acids, and Gly have been also involved in the antioxidant activity of protein hydrolysates [20,22,23,24,25]. From multiple sequence alignment of the amino acid sequences, it can be assumed that the main sheep plasma proteins are rich in hydrophobic, basic, and acidic amino acids (from 62% to 67% in the case of the fibrinogen depending on the specific chain, up to 80% in serum albumin). According to Wu et al. [17] the content of hydrophobic, basic, and acidic amino acids is more than 80% in sheep plasma. However, due to the complex nature of Alcalase and lacking the knowledge of enzyme cutting sites, it is not possible to predict the peptides in Alcalase-hydrolysates turning to predictive computing. Furthermore, there are few studies on the preparation, isolation, and purification of antioxidant peptides obtained from sheep blood plasma, with the specific peptides mainly implied not been yet identified.

The current study aimed at isolating and identifying the peptides with better antioxidant properties obtained from Alcalase-hydrolysis of sheep plasma using semi-preparation reverse-phase high-performance liquid chromatography (RP-HPLC), AKTA protein rapid separation and purification system, and liquid chromatography-tandem mass spectrometry (LC-MS/MS).

## 2. Materials and Methods

### 2.1. Materials

Fresh sheep blood (5 L) was obtained from a local slaughterhouse in Beijing (China). Sodium citrate (0.5% w/v final concentration) was added just after collection to prevent coagulation and transported to the laboratory under refrigerated conditions (4–6 °C). Plasma was obtained by decanting after centrifugation at 8000× *g* for 20 min at 4 °C (Himac CR22 GII, Hitachi, Ltd., Tokyo, Japan) and frozen at −80 °C for 4 h before being lyophilized (LGJ-25; Four-Ring Science Instrument Plant Beijing Co., Ltd., Beijing, China). The antioxidant activity (capacity of ferric-reducing antioxidant power (FRAP) and the scavenging ability of 2,2-diphenyl-1-picryl-hydrazyl-hydrate (DPPH) free radical of the dehydrated plasma was determined before storage and then frozen at −20 °C until its submission to enzymatic hydrolysis. 

### 2.2. Enzymatic Hydrolysis of Sheep Plasma

SPPH was prepared in triplicate as described by Wu et al. [17], with only some small modifications. In brief, sheep plasma powder was dissolved in 0.01 M phosphate buffered saline (PBS, pH 7) to a final concentration of 40 mg/mL. Alcalase (EC: 3.4.21.62, 200 000 U/g, Solarbio, Beijing, China) was added at 5000 U/g protein to reconstituted plasma solution (125 mL). The enzymatic hydrolysis was carried out at 55 °C and pH 11 for 6 h. After that, the reaction was stopped by enzyme inactivation at 90 °C for 10 min and the hydrolysate pH adjusted to 7 before being centrifuged at 8000× *g* for 15 min under refrigeration conditions. The supernatant was collected, freeze-dried and stored at −20 °C for further uses. The obtained powdered SPPHs were mixed together and the antioxidant activity of the mixture was determined before starting the fractionation step. The protein/peptide sample concentrations were measured using a modified Lowry protein assay kit (Thermo Scientific, California, USA).

### 2.3. Fractionation of Hydrolysates by Ultrafiltration Separation

A centrifugal-ultrafiltration based-method was applied for isolating peptide fractions from the SPPH by using tubes containing centrifugal filters devices, which differed in their molar mass selectivity. Specifically, Amicon^®^ Ultra-15 10 K and 3 K filter devices (Millipore, Billerica, MA) with molecular weight cut-offs (MWCO) of 10 and 3 kDa, respectively, and Vivaspin^®^ Turbo centrifugal concentrators of 5 kDa MWCO (Sartorius, Goettingen, Germany) were used to obtain sequentially 4 different peptide fractions in terms of MW (>10 kDa, 5–10 kDa, 3–5 kDa and <3 kDa). Firstly, the tube containing the 10 kDa MWCO filter device was loaded with 10 mL of the hydrolysate solution (40 mg/mL) prepared from SPPH powder and distilled water. After centrifugation at 3000× *g* and 4 °C for 20 min, the resulting retentate (>10 kDa fraction) was resuspended in distilled water before being recovered for subsequent freeze-drying. Furthermore, the filtrate was collected, loaded into the 5 kDa MWCO tube, and centrifuged again. The same procedure just indicated above was newly applied to both the retentate and the filtrate containing peptides with a 5–10 kDa MW and <5 kDa, respectively, submitting this last fraction to a new separation step to obtain two more peptide fractions, with 3-5 kDa and <3 kDa MWs, respectively. The antioxidant activity of all fractions obtained after the ultrafiltration process was determined and then they were freeze-dried. 

### 2.4. Fractionation by Semi-Preparation Reverse-Phase High-Performance Liquid Chromatography

Semi-preparation RP-HPLC according to Hernández-Ledesma et al. [26] with slight modifications was used for isolation and purification of sheep plasma peptides fraction with the highest antioxidant activity (<5 kDa MW). The peptide fraction was dissolved in 50 mM PBS at pH 7 to a final concentration of 40 mg/mL and the peptides separation was performed at room temperature with an Innoval C_18_ column (Agilent Technologies, Santa Clara, CA, USA), with the following characteristics: 10 μm particle size, 100 Å pore size, and 21.2 × 250 mm I.D. A Varian LC system (Agilent Prostar 218, Agilent Technologies) equipped with Prostar 210/218 pumps, a Prostar 325 UV-Vis detector, a 440-LC fraction collector and a Rheodyne 7725i injector allowing injection of 1 mL sample was used to carry out the peptide separation. The mobile phase A was ultrapure water containing 0.1% trifluoroacetic acid (TFA) and the mobile phase B was acetonitrile with 0.1% TFA. The peptides were eluted at a flow-rate of 10 mL/min following a linear gradient by increasing mobile phase B from 5% to 20% in 30 min and held at this percentage for an additional 5 min. The column was washed for 10 min between injections with a mobile phase with the same composition as at the start of the separation run. The detection wavelength was set at 220 nm. Nine fractions (F1–F9) were manually collected from nine runs and lyophilized. The antioxidant activity was determined for each fraction. To be clear, a very small percentage of peptides will be able to form TFA-salt when lyophilized. To minimize this problem, the peptide fractions were compared at the same concentration.

### 2.5. Fractionation by AKTA Protein Rapid Separation and Purification System

The RP-HPLC fractions with the highest antioxidant activity were redissolved in distilled water to a final concentration of 10 mg/mL and purified by size exclusion chromatography using an ÄKTA^TM^ Pure protein purification system (GE Healthcare Bio-Sciences AB, Uppsala, Sweden) equipped with a Superdex^®^ Peptide 10/300 GL column (GE Healthcare), with a 30 cm × 10 mm (L × D) dimension and 13 µm average particle size. Purification conditions were: mobile phase, 50 mM PBS at pH 7; flow rate, 0.5 mL/min; column volume, 1.3 CV; and detection wavelength, 214 nm. According to the chromatogram, four (F1-1, F1-2, F1-3, and F1-4) and five (F2-1, F2-2, F2-3, F2-4, and F2-5) fractions were manually collected from F1 and F2, respectively, and lyophilized. The antioxidant activity was determined for each fraction after reconstitution with distilled water (100 μg/mL).

### 2.6. Determination of Antioxidant Properties

Dehydrated sheep plasma, sheep plasma protein hydrolysate and the purified peptide fractions coming from the hydrolysate were fully dissolved in distilled water at 100 μg/mL and their antioxidant properties were measured by the FRAP and the DPPH scavenging assays. 

The FRAP assay, which estimates the total antioxidant capacity by measuring the reduction of ferric-tripyridyltriazine complex to ferrous-tripyridyltriazine [27], was determined using analytical kits purchased from Nanjing Jiancheng Bioengineering Institute (Nanjing, China). The assay was performed according to the manufacturer’s instructions. The absorbance of the samples measured at 593 nm using a spectrophotometer CM-600D (Konica Minolta Sensing Americas Inc., Tokyo, Japan) was used to calculate the FRAP value according to the method described by Bolanos de la Torre et al. [28]. Each sample was analyzed in triplicate.

The DPPH assay was used to determine the scavenging capacity of the different samples. This method is based on using DPPH as a stable and colored free radical and determining by spectrophotometry the formation of reduced DPPH in the presence of antioxidant compounds in the sample [29]. The measurement was performed according to You et al. [30] with some modifications. 0.1 mM DPPH was prepared in 95% ethanol, which showed a deep violet color. The absorbance value at 517 nm of different mixtures (A_S0_, A_S1_, A_S2_, and A_S3_) was recorded after incubating all of them for 20 min in dark at room temperature. S0 corresponds to the solution prepared by mixing 1.5 mL distilled water and 1.5 mL 95% ethanol. S1 and S2 were mixtures obtained by adding 1.5 mL 0.1 mM DPPH solution and 1.5 mL 95% ethanol, respectively, to 1.5 mL sample. Finally, in S3 1.5 mL distilled water was mixed in 1.5 mL 0.1 mM DPPH solution. All measurements were made in duplicate. The radical scavenging rate was calculated from the decrease in the absorbance by using the following equation:

DPPH radical scavenging rate (%) = [1 − (A_S1_ − As_2_)/(As_3_ − As_0_)] × 100

where A_S0_ was the absorbance of ethanol solution; A_S1_ and A_S2_ was the absorbance of the test sample mixed with DPPH solution and ethanol, respectively; A_S3_ was the absorbance of DPPH solution.

### 2.7. Peptide Identification by LC-MS/MS

The peptide compounds of the two most active fractions were identified by LC-MS/MS according to Le Maux et al. [31] with minor modifications. Each fraction was loaded on a pre-column (EASY-Column^TM^ C_18_, 100 μm × 2 cm, 5 μm particle size, Thermo Scientific, Waltham, Massachusetts, USA) and separated on a capillary column (EASY-Column^TM^ C_18_, 75 μm × 10 cm, 3 μm particle size, Thermo Scientific) with a mobile buffer A (0.1% formic acid) and B (84% acetonitrile and 0.1% formic acid) at a flow rate of 300 nL/min. The linear gradient was performed as follows: 0–35% buffer B for 50 min, 35–100% buffer B for 5 min, 100% buffer B for 5 min. 

LC-MS/MS analysis was performed on a Q-Exactive mass spectrometer (Thermo Scientific), and the spectra were searched using MASCOT engine (version 2.4; Matrix Science, London, UK). The mass spectrometer was operated in positive ion mode, and a full MS survey scans were performed using a mass range of 300–1800 m/z. High-energy collision dissociation (HCD) fragmentation was used for MS/MS and the twenty most intense signals in the survey scan were fragmented. Automatic gain control (AGC) target was set to 1  ×  10^6^, and maximum inject time to 40 ms. Dynamic exclusion duration was 60 s. Survey scans were acquired at a resolution of 70,000 at 200 m/z and resolution for HCD spectra was set to 17,500 at 200 m/z, and isolation width was 2 m/z. The normalized collision energy was 30 eV and the underfill ratio, which specifies the minimum percentage of the target value likely to be reached at maximum fill time, was defined as 0.1%. The molecular weight (MW) and theoretical isoelectric point (pI) of the peptides were calculated by the Compute pI/MW tool (https://web.expasy.org/compute_pi/).

### 2.8. Statistical Analysis

Data are presented as mean ± standard deviation from three independent replicate determinations. The statistical analyses were performed using the SAS 9.2 statistical package (SAS Institute, Cary, NC, USA). The effect of Alcalase-hydrolysis on the antioxidant properties of sheep plasma was examined using the unpaired Student’s two-tailed *t*-test between sheep plasma and Alcalase-derived hydrolysate. A one-way analysis of variance (ANOVA) was applied when comparing the antioxidant properties of the different protein fractions and, when appropriate, differences among the mean values were established using the least significant difference (LSD) test. In any case, differences were considered significant when *p* < 0.05.

## 3. Results and Discussion

### 3.1. Antioxidant Properties of Alcalase-Hydrolysate Prepared from Sheep Plasma

In this study, the antioxidant properties of all samples were assessed by two different methods, the FRAP antioxidant capacity and the DPPH radical scavenging ability assays, because of the big range of antioxidant molecules in plasma and, consequently, the different mechanisms implied in the antioxidant capacity; thus, a single assay may not reflect the true antioxidant potential [32,33]. Plasma antioxidants include some enzymes acting as radical scavengers [34], but also many nonenzymatic compounds with different characteristics, including high-MW compounds like albumin or transferrin and low-MW antioxidants as ascorbic acid, tocopherol, and glutathione, among others [35]. FRAP values could be proportional to the reducing power of these last ones [36].

**Figure 1:**
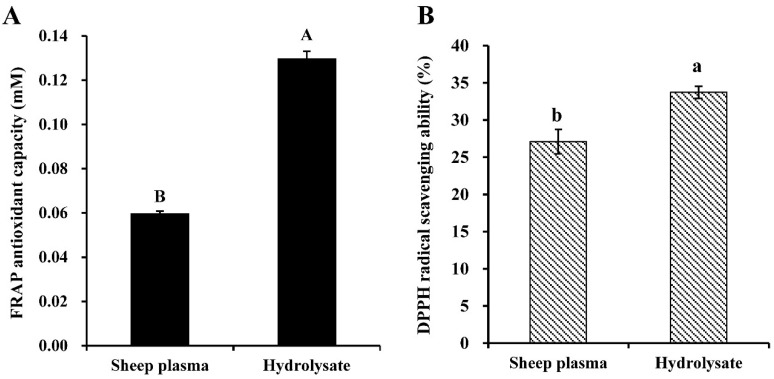
Ferric-reducing antioxidant power (FRAP) (**A**) and the 2,2-diphenyl-1-picrylhydrazyl (DPPH) radical scavenging ability (**B**) of Alcalase-hydrolysate prepared from sheep plasma. The concentration of the sample is 5 mg/mL. Different letters indicate significant differences between sheep plasma and hydrolyzate by unpaired Student’s t-test (capital letters (A and B): for α = 0.01; lower case letters (a and b): for α = 0.05). Means ± SD (n = 3).

FRAP antioxidant capacity of sheep plasma was 598 ± 10 mM FeSO_4_ equivalent per g of sample (Figure 1). This result would agree with Bah et al. [3], who reported similar levels of FRAP activity for deer, sheep and cattle plasma but a higher activity in the pig plasma. It has been estimated that the relative contribution of plasma proteins to the total FRAP value is only 10% [36]. Proteins, lipids, and vitamins, among others, can contribute in different degrees to the antioxidant potential of plasma [36,37,38].

DPPH radical scavenging activity of sheep plasma was 27.09 ± 1.63% (Figure 1), which was consistent with the results of Bah et al. [3]. Moreover, they found that sheep plasma had the highest DPPH radical scavenging activity (27.6 ± 2.0%) compared to deer (21.9 ± 2.2%), cattle (21.3 ± 1.5%), and pig (17.4 ± 4.7%).

The antioxidant power of sheep plasma proteins could be enhanced after their enzymatic treatment with different proteases because of the higher exposure of functional amino acid residues in the peptide hydrolysate [39,40]. As can be seen in Figure 1, Alcalase-treatment of sheep plasma under the conditions established in the present work significantly (*p* < 0.05) improved its antioxidant properties, but the increment was depending on the specific measured parameter. After a 6 h-hydrolysis, the DPPH radical scavenging activity of sheep plasma was increased by around 24%, reaching a value of 33.72 ± 0.82%, which can be considered relatively high compared to that obtained after hydrolysis using a FPII protease for 24 h (63.3%) [39]. Although it is well established that the effects of the enzymatic treatment depend strongly on the enzyme used and the hydrolysis time, the results obtained also depend on the initial protein substrate. In this sense, it has been shown that pig plasma hydrolysate obtained after being treated with Alcalase for 5 h exhibited a relatively high free radical scavenging activity (76.5 ± 1.5%) compared to sheep plasma [41], while in the case of a 24 h-trypsin hydrolysate was only 11.5 ± 2.3% [42]. The animal origin of plasma determines the particular amino acid sequence of its proteins. In the case of sheep and pig, serum albumin and fibrinogen -two of the three most abundant plasma proteins- have only a 69–84% similarity; thus, these differences may be partially responsible for the discrepancies in antioxidant ability.

Moreover, after the 24 h-Alcalase-hydrolysis, antioxidant capacity, as measured by FRAP, was increased by 116.67%. Alcalase-pig plasma hydrolysate also showed increased FRAP values (1.56–3.08-fold) compared to the untreated plasma protein, depending on the hydrolysis degree within the range from 6.2% to 17.6% [41]. 

### 3.2. Antioxidant Properties of Peptide Fractions Obtained by Ultrafiltration

Ultrafiltration, RP-HPLC, and LC-MS/MS have been often used to isolate and identify peptides with good antioxidant properties [41,43].

**Figure 2:**
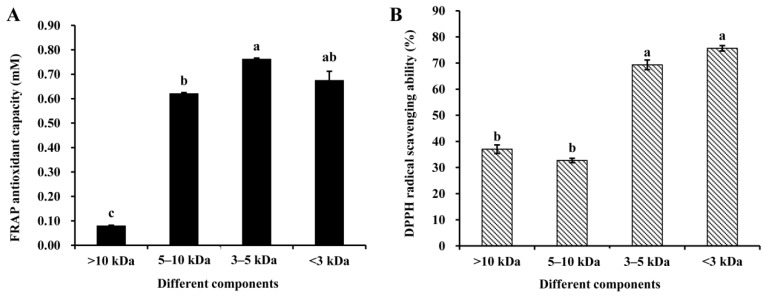
Ferric-reducing antioxidant power (FRAP) (**A**) and the 2,2-diphenyl-1-picrylhydrazyl (DPPH) radical scavenging ability (**B**) of peptide fractions obtained by ultrafiltration. The concentration of the sample is 5 mg/mL. Different letters(a–c) indicate significant differences among treatments for α = 0.05. Means ± SD (n = 3).

In the present study, the antioxidant properties of peptide fractions obtained by ultrafiltration are presented in Figure 2. Figure 2A shows the FRAP antioxidant capacity, while DPPH the radical scavenging ability is shown in Figure 2B. As can be observed, the FRAP value shows a great and significant (*p* < 0.05) increase when the peptide’s MW was reduced below 10 kDa, being more than 3.8-fold and 9.4-fold higher than that of unpurified hydrolysate and untreated sheep plasma, respectively. The fraction containing 3–5 kDa MW peptides presented the highest antioxidant capacity, but without significantly (*p* > 0.05) differing from the fraction consisting of peptides with a MW < 3 kDa. These results contrast with the behavior shown by bambara groundnut Alcalase-protein hydrolysates, with the ferric reducing power been practically independent of MW [19].

The DPPH radical scavenging ability also depends on the peptide’s molecular size. In Figure 2B, it can be clearly observed that there was a strong and significant (*p* < 0.05) increase in this activity when the peptide MW is <5 kDa. DPPH radical scavenging ability of fractions of MW < 3 kDa (75.65 ± 1.83%) and 3–5 kDa (69.31 ± 2.24%) was significantly (*p* < 0.05) higher than those of MW 5–10 kDa (32.71 ± 1.02%) and >10 kDa (37.03 ± 1.83%). At this point, it is important to note that, in spite of no significant differences (*p* > 0.05) being observed between 3–5 kDa and <3 kDa fractions, the mean value tended to be higher in this last one. Therefore, this behavior would be partially consistent with that reported by Arise et al. [19] for peptide’s fractions obtained from an Alcalase-protein hydrolysate of bambara groundnut. They also observed that peptides with low MW were more effective radical scavengers than larger ones working in a similar range of MWs, and according to them, the higher DPPH radical activity could be related to their lower surface hydrophobicity. Liu et al. [41] also observed a similar relationship among the degree of Alcalase-hydrolysis of porcine plasma proteins, DPPH scavenging ability and surface hydrophobicity, and suggested the greater structural flexibility of the peptides in relation to the native protein as a key point. By contrast, Ajibola et al. [44] observed surface hydrophobicity increased as MW decreased, although a similar behavior was found when analyzing the relationship between radical scavenger activity and MW of Alcalase-protein hydrolysate. 

Taking into account that the antioxidant activities of 3–5 kDa and <3 kDa fractions were not significantly different (*p* > 0.05) between them, the <5 kDa fraction was chosen for further isolation and purification of the antioxidative peptide. It would be in good agreement with those from other studies showing that peptides in this MW range play an important role in antioxidant activity [41,45,46,47,48,49].

### 3.3. Purification of Antioxidant Peptides by Semi-Preparation RP-HPLC

**Figure 3:**
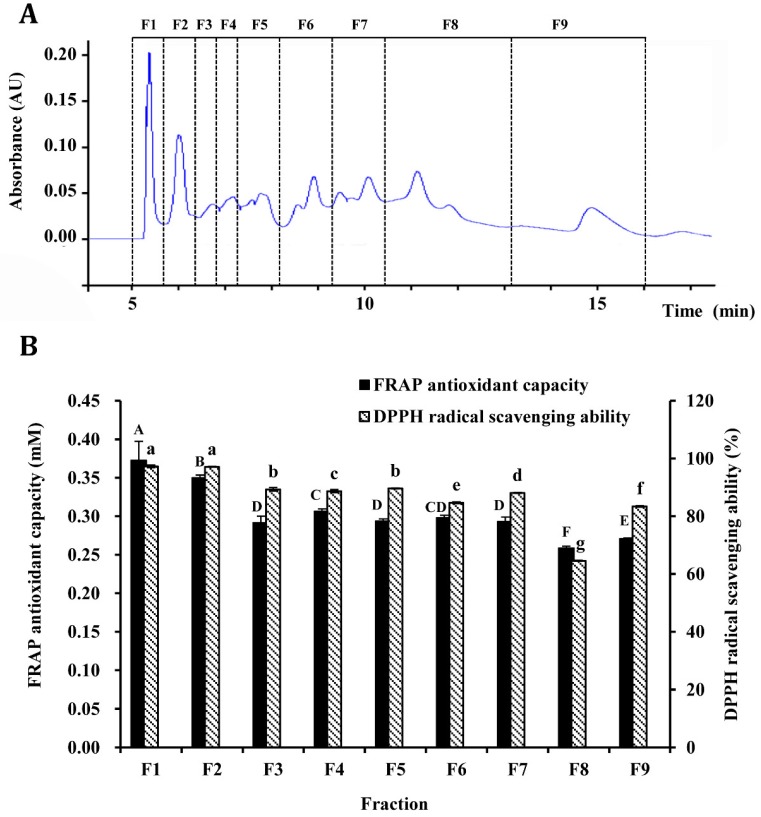
(**A**) Elution diagram of sheep plasma protein hydrolysate with molecular weights <5 kDa by semi-preparation reverse-phase high-performance liquid chromatography (RP-HPLC). (**B**) Ferric-reducing antioxidant power (FRAP) and the 2,2-diphenyl-1-picrylhydrazyl (DPPH) radical scavenging ability of the fractions (F1–F9) purified by semi-preparation RP-HPLC. The concentration of the sample is 100 μg/mL. Different letters (A–F, a–g) in the same column indicate significant difference among treatments for α = 0.05. Means ± SD (n = 3).

The <5 kDa fraction was further purified by semi-preparation RP-HPLC, and nine fractions (F1–F9) were obtained based on retention time (Figure 3A). The FRAP antioxidant capacity and DPPH radical scavenging ability of these fractions were evaluated (Figure 3B). As can be observed, F1 fraction displayed the significantly (*p* < 0.05) highest FRAP activity, while a significantly higher (*p* < 0.05) DPPH radical scavenging ability was found in both F1 (97.28%) and F2 (97.10%) fractions as compared to other fractions (64.58–89.58%).

Several studies focused on determining the biological activity of protein hydrolysates, which were obtained from different raw materials and using different proteolytic enzymes, have shown that their antioxidant activity is closely related to the size of peptides, with the most relevant contribution corresponding to the <1 kDa fraction. However, some more recent studies also point out as other key factors the richness in aromatic and hydrophobic amino acids and the structure of these peptides [44,48,50,51,52]. In order to elucidate whether F1 and F2 fractions are rich in small MW peptides, they must first be purified.

### 3.4. Purification of Antioxidant Peptides by AKTA Protein Rapid Separation and Purification System

**Figure 4:**
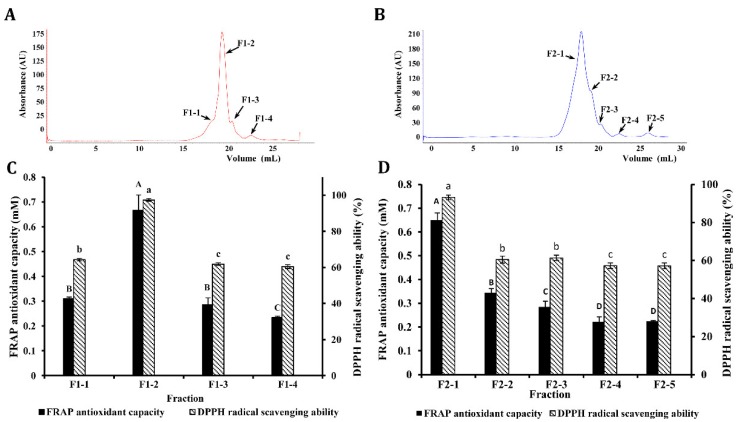
Elution diagram of the F1 (**A**) and F2 (**B**) fractions by AKTA protein rapid separation system. Ferric-reducing antioxidant power (FRAP) and the 2,2-diphenyl-1-picrylhydrazyl (DPPH) radical scavenging ability of the separations of the F1 fraction (**C**) and F2 fraction (**D**). The concentration of the sample is 100 μg/mL. Different letters (A–D, a–c) in the same column indicate significant difference among treatments for α = 0.05. Means ± SD (n = 3).

The F1 and F2 fractions were further separated by the AKTA purifier system using the Superdex Peptide 10/300 GL column to generate four (F1-1–F1-4) and five (F2-1–F2-5) fractions, respectively (Figure 4A). The FRAP antioxidant capacity and DPPH radical scavenging ability of these fractions are shown in Figure 4C,D.

F1-2 and F2-1 fractions exhibited the significantly (*p* < 0.05) highest FRAP and DPPH radical scavenging activities (Figure 4C,D), respectively, with values markedly higher than those of the other fractions. Both fractions were selected for peptide sequence identification

### 3.5. Peptide Characterization and Identification of Their Sequence

**Figure 5:**
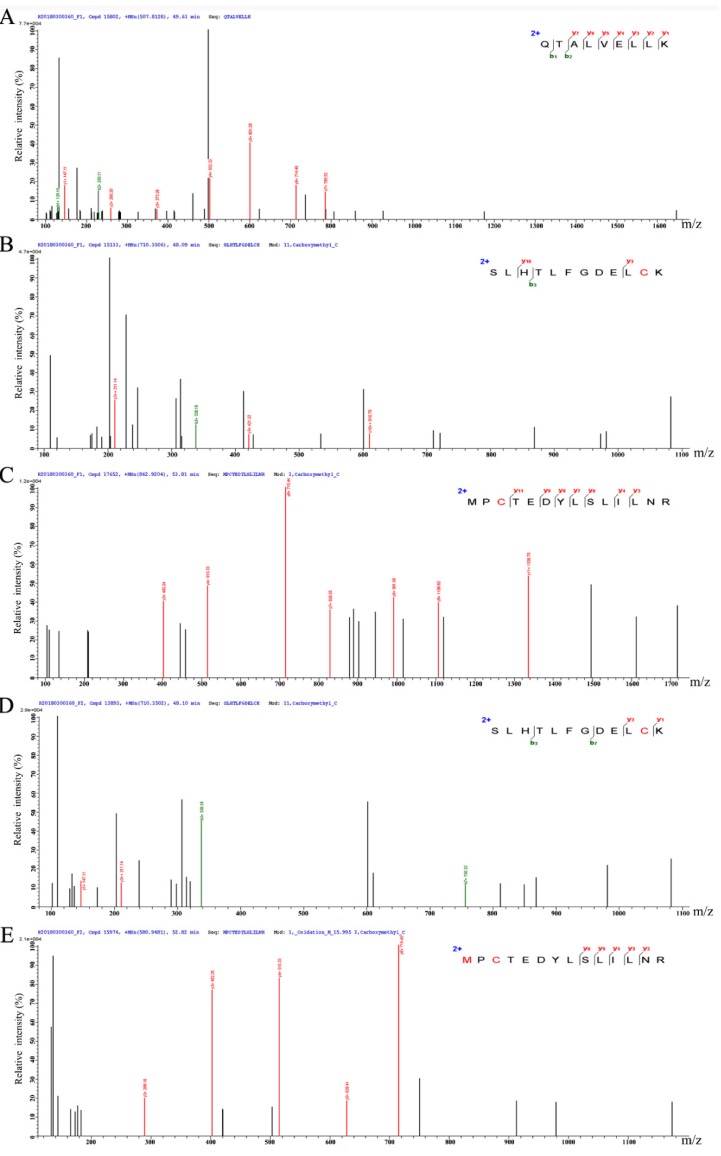
The amino acid sequence of antioxidative peptide identified from F1-2 fraction (**A–C**) and F2-1 fraction (**D**,**E**). (**A**) A peptide with the sequence of QTALVELLK was identified from ion *m/z* 507.81. (**B**) A peptide with the sequence of SLHTLFGDELCK was identified from ion *m/z* 710.35. (**C**) A peptide with the sequence of MPCTEDYLSLILNR was identified from ion *m/z* 862.92. (**D**) A peptide with the sequence of SLHTLFGDELCK was identified from ion *m/z* 710.35. (**E**) A peptide with the sequence of MPCTEDYLSLILNR was identified from ion *m/z* 580.95.

Using LC-MS/MS, three predominant peptides composed of 9–14 amino acid residues were found from F1-2 and F2-1 (Table 1), whose MW ranges from 1014.23 to 1667.96 Da, and their pI from 4.37 to 6.00. All these peptides were identified in F1-2 fraction, while only two in F2-1 fraction. The amino acid sequence of purified peptide was shown in Figure 5.

According to literature, peptides consisting of arginine (R), lysine (K), histidine (H), glutamine (Q), aspartic acid (D), methionine (M), tyrosine (Y), valine (V), phenylalanine (F), leucine (L), cysteine (C), and/or proline (P) have been reported to possess a strong antioxidant activity [20,23,24]. Aspartic acid and glutamine exhibit free radical quenching activity due to the presence of excess electron, histidine display strong radical scavenging activity due to the presence of an imidazole ring as an important proton donor, the pyrrolidine and indole ring in proline and tryptophan, respectively, could also serve as hydrogen donors via their hydroxyl groups, thus acting as hydroxyl radical scavengers [53]. The hydrophobic amino acids (i.e., valine, leucine, and phenylalanine) may be responsible for forming a favorable hydrophobic micro-environment for peptide molecules [53]. Previous studies also indicated that the amino group of basic amino acids, the carboxyl group of acidic amino acids, and the phosphorylated serine residues play an important role in the chelation of metal ions [23,54]. Moreover, it is important to take into account the amino acid sequence of the isolated peptides. According to Saiga et al. [55], the primary structure of peptides can also affect the antioxidant activity. As it can be observed from the sequence reported in Table 1, two of them were identified in both fractions. As a whole, it can be maintained that several amino acids identified in the isolated Alcalase-protein hydrolysates from sheep plasma might be responsible for their oxidative stability. Moreover, the C-ends of identified antioxidative peptides were lysine (K) and arginine (R), which was similar to the reported by other researchers for antioxidant peptides [20,23], and some also consisted of one or two acidic amino acid residue (D, E) and basic amino acid residue (K, R, and H).

It is important to note that the Basic Local Alignment Search Tool (BLAST) analysis showed that the amino acid sequence of these peptide segments is highly homologous to the primary structure of sheep serum albumin. This would be in good agreement with the authors maintaining that albumin is a major circulating extracellular antioxidant [56,57]. 

## 4. Conclusions

In summary, three peptides were isolated and characterized from different fractions coming from the Alcalase-protein hydrolysate using sheep plasma as a raw material, which exhibited appreciable FRAP activity and DPPH radical scavenging activity. Their sequences (QTALVELLK, SLHTLFGDELCK and MPCTEDYLSLILNR) include amino acids that have been previously reported as key contributors to the peptide antioxidant properties. Thus, the sheep plasma can be considered a good source of antioxidant peptides that might be a useful food additive and a bioactive material, and bring forth new possibilities for utilization of animal blood by-products. Moreover, these peptides seem to come mainly from serum albumin. In this context, its isolation and subsequent Alcalase-hydrolysis could be considered as a strategy to obtain more easily the peptides of interest and, probably, to decrease the economical cost. Further studies should be done to determine the physiological functions, structure-activity relationship and application of the identified peptides. 

## Figures and Tables

**Table 1:** Peptides identified by liquid chromatography-tandem mass spectrometry in F1-2 and F2-1 fraction.

Fraction	Sequence	MW (Da)	pI	Special Amino Acids Ratio (%)		
				Hydrophobic Amino Acids	Acid Amino Acids	Basic Amino Acid
F1-2	QTALVELLK	1014.23	6.00	55.56	11.11	11.11
	SLHTLFGDELCK	1362.56	5.30	33.33	16.67	16.67
	MPCTEDYLSLILNR	1667.96	4.37	50.00	14.29	7.14
F2-1	SLHTLFGDELCK	1362.56	5.30	33.33	16.67	16.67
	MPCTEDYLSLILNR	1667.96	4.37	50.00	14.29	7.14

## References

[1] Chen Y., Zhu W., Chen Z. (2018). The determinants of mutton consumption-at-home in urban China using an IHS double-hurdle model. Br. Food J..

[2] National Bureau of Statistics of China. http://data.stats.gov.cn/english/.

[3] Bah C.S., Bekhit A.E.-D.A., Carne A., McConnell M.A. (2016). Composition and biological activities of slaughterhouse blood from red deer, sheep, pig and cattle. J. Sci. Food Agric..

[4] Sorapukdee S., Narunatsopanon S. (2017). Comparative study on compositions and functional properties of porcine, chicken and duck blood. Korean J. Food Sci. Anim. Resour..

[5] Verma A.K., Chatli M.K., Mehta N., Kumar P. (2018). Assessment of physico-chemical, antioxidant and antimicrobial activity of porcine blood protein hydrolysate in pork emulsion stored under aerobic packaging condition at 4 ± 1 °C. Lwt Food Sci. Technol..

[6] Del Hoyo P., Rendueles M., Díaz M. (2008). Effect of processing on functional properties of animal blood plasma. Meat Sci..

[7] Ofori J.A., Hsieh Y.H.P., El-Samragy Y. (2012). The Use of Blood and Derived Products as Food Additives.

[8] García M.C., Puchalska P., Esteve C., Marina M.L. (2013). Vegetable foods: A cheap source of proteins and peptides with antihypertensive, antioxidant, and other less occurrence bioactivities. Talanta.

[9] He R., Malomo S.A., Alashi A., Girgih A.T., Ju X., Aluko R.E. (2013). Purification and hypotensive activity of rapeseed protein-derived renin and angiotensin converting enzyme inhibitory peptides. J. Funct. Foods.

[10] Thammarat K., Leena N., Punnanee S., Soottawat B. (2015). Functional and antioxidative properties of Bambara groundnut (*Voandzeia subterranea*) protein hydrolysates. Int. Food Res. J..

[11] Wu L., Hou C., Zhao M., Zhang D. (2018). Preparation and process optimization of sheep plasma protein antioxidant peptide. Food Sci. Technol..

[12] Guimarães Drummond e Silva F., Miralles B., Hernández-Ledesma B., Amigo L., Iglesias A.H., Reyes Reyes F.G., Netto F.M. (2017). Influence of protein–phenolic complex on the antioxidant capacity of flaxseed (*Linum usitatissimum* L.) products. J. Agric. Food Chem..

[13] Mamelona J., Saint-Louis R., Pelletier É. (2010). Nutritional composition and antioxidant properties of protein hydrolysates prepared from echinoderm byproducts. Int. J. Food Sci. Technol..

[14] Peng X., Xiong Y.L., Kong B. (2009). Antioxidant activity of peptide fractions from whey protein hydrolysates as measured by electron spin resonance. Food Chem..

[15] Silva F.G.D.e., Hernández-Ledesma B., Amigo L., Netto F.M., Miralles B. (2017). Identification of peptides released from flaxseed (*Linum usitatissimum*) protein by Alcalase^®^ hydrolysis: Antioxidant activity. Lwt Food Sci. Technol..

[16] Delange R.J., Smith E.L. (1968). Subtilisin Carlsberg. I. Amino acid composition; isolation and composition of peptides from the tryptic hydrolysate. J. Biol. Chem..

[17] Wu L., Hou C., Xi B., Aude Ingrid Boga L., Zhang D. (2018). Sheep plasma hydrolysate inhibits lipid and protein oxidation to improve color stability in mutton patties. Food Sci. Technol. Res..

[18] Ryan J.T., Ross R.P., Bolton D., Fitzgerald G.F., Stanton C. (2011). Bioactive peptides from muscle sources: Meat and fish. Nutrients.

[19] Arise A.K., Alashi A.M., Nwachukwu I.D., Ijabadeniyi O.A., Aluko R.E., Amonsou E.O. (2016). Antioxidant activities of bambara groundnut (*Vigna subterranea*) protein hydrolysates and their membrane ultrafiltration fractions. Food Funct..

[20] Ma Y., Wu Y., Li L. (2018). Relationship between primary structure or spatial conformation and functional activity of antioxidant peptides from *Pinctada fucata*. Food Chem..

[21] Wattanasiritham L., Theerakulkait C., Wickramasekara S., Maier C.S., Stevens J.F. (2016). Isolation and identification of antioxidant peptides from enzymatically hydrolyzed rice bran protein. Food Chem..

[22] Je J.-Y., Lee K.-H., Lee M.H., Ahn C.-B. (2009). Antioxidant and antihypertensive protein hydrolysates produced from tuna liver by enzymatic hydrolysis. Food Res. Int..

[23] Sabeena Farvin K.H., Baron C.P., Nielsen N.S., Otte J., Jacobsen C. (2010). Antioxidant activity of yoghurt peptides: Part 2—Characterisation of peptide fractions. Food Chem..

[24] Sah B.N.P., Vasiljevic T., McKechnie S., Donkor O.N. (2014). Effect of probiotics on antioxidant and antimutagenic activities of crude peptide extract from yogurt. Food Chem..

[25] Wu Y.Y., Li L.H., Duan Z.H., Yang X.Q., Shang J., Chen S.J. (2012). Application of response surface methodology to optimise preparation high antioxidant activity product from pinctada fucata muscle. Adv. Mater. Res..

[26] Hernández-Ledesma B., Dávalos A., Bartolomé B., Amigo L. (2005). Preparation of antioxidant enzymatic hydrolysates from α-lactalbumin and β-lactoglobulin. Identification of active peptides by HPLC-MS/MS. J. Agric. Food Chem..

[27] Irshad M., Ahmad I., Mehdi S.J., Goel H.C., Rizvi M.M.A. (2014). Antioxidant capacity and phenolic content of the aqueous extract of commonly consumed cucurbits. Int. J. Food Prop..

[28] Bolanos de la Torre A.A.S., Henderson T., Nigam P.S., Owusu-Apenten R.K. (2015). A universally calibrated microplate ferric reducing antioxidant power (FRAP) assay for foods and applications to Manuka honey. Food Chem..

[29] Huang D., Ou B., Prior R.L. (2005). The chemistry behind antioxidant capacity assays. J. Agric. Food Chem..

[30] You L., Zhao M., Regenstein J.M., Ren J. (2011). In vitro antioxidant activity and in vivo anti-fatigue effect of loach (*Misgurnus anguillicaudatus*) peptides prepared by papain digestion. Food Chem..

[31] Le Maux S., Nongonierma A.B., Murray B., Kelly P.M., FitzGerald R.J. (2015). Identification of short peptide sequences in the nanofiltration permeate of a bioactive whey protein hydrolysate. Food Res. Int..

[32] Dontha S. (2016). A review on antioxidant methods. Asian J. Pharm. Clin. Res..

[33] Zheng L., Lin L., Su G., Zhao Q., Zhao M. (2015). Pitfalls of using 1,1-diphenyl-2-picrylhydrazyl (DPPH) assay to assess the radical scavenging activity of peptides: Its susceptibility to interference and low reactivity towards peptides. Food Res. Int..

[34] Möller J., Gilman J.T., Sussmane J., Raszynski A., Wolfsdorf J. (1993). Changes in plasma levels of oxygen radical scavenging enzymes during extracorporeal membrane oxygenation in a lamb model. Biol. Neonate.

[35] Ďuračková Z. (2010). Some current insights into oxidative stress. Physiol. Res..

[36] Benzie I.F.F., Strain J.J. (1996). The ferric reducing ability of plasma (FRAP) as a measure of “antioxidant power”: The FRAP assay. Anal. Biochem..

[37] Niki E. (1987). Lipid antioxidants: How they may act in biological systems. Br. J. Cancer Suppl..

[38] Esterbauer H., Gebicki J., Puhl H., Jürgens G. (1992). The role of lipid peroxidation and antioxidants in oxidative modification of LDL. Free Radic. Biol. Med..

[39] Bah C.S.F., Bekhit A.E.-D.A., Carne A., McConnell M.A. (2015). Production of bioactive peptide hydrolysates from deer, sheep and pig plasma using plant and fungal protease preparations. Food Chem..

[40] Kong B., Xiong Y.L. (2006). Antioxidant activity of zein hydrolysates in a liposome system and the possible mode of action. J. Agric. Food Chem..

[41] Liu Q., Kong B., Xiong Y.L., Xia X. (2010). Antioxidant activity and functional properties of porcine plasma protein hydrolysate as influenced by the degree of hydrolysis. Food Chem..

[42] Wei J.T., Beenhuang C. (2009). Bioactive peptide production by hydrolysis of porcine blood proteins in a continuous enzymatic membrane reactor. J. Sci. Food Agric..

[43] Bah C.S.F., Bekhit A.E.-D.A., McConnell M.A., Carne A. (2016). Generation of bioactive peptide hydrolysates from cattle plasma using plant and fungal proteases. Food Chem..

[44] Ajibola C.F., Fashakin J.B., Fagbemi T.N., Aluko R.E. (2011). Effect of peptide size on antioxidant properties of African yam bean seed (*Sphenostylis stenocarpa*) protein hydrolysate fractions. Int. J. Mol. Sci..

[45] He R., Girgih A.T., Malomo S.A., Ju X., Aluko R.E. (2013). Antioxidant activities of enzymatic rapeseed protein hydrolysates and the membrane ultrafiltration fractions. J. Funct. Foods.

[46] Hu F., Ci A.T., Wang H., Zhang Y.Y., Zhang J.G., Thakur K., Wei Z.J. (2018). Identification and hydrolysis kinetic of a novel antioxidant peptide from pecan meal using Alcalase. Food Chem..

[47] Park P.J., Jung W.K., Nam K.S., Shahidi F., Kim S.K. (2001). Purification and characterization of antioxidative peptides from protein hydrolysate of lecithin-free egg yolk. J. Am. Oil Chem. Soc..

[48] Sonklin C., Laohakunjit N., Kerdchoechuen O. (2018). Assessment of antioxidant properties of membrane ultrafiltration peptides from mungbean meal protein hydrolysates. PeerJ.

[49] Yu P., Linden D.S.v.d., Sugiarto H., Anderson R.C. (2010). Antimicrobial peptides isolated from the blood of farm animals. Anim. Prod. Sci..

[50] Chai H.J., Chan Y.L., Li T.L., Shiau C.Y., Wu C.J. (2013). Evaluation of lanternfish (*Benthosema pterotum*) hydrolysates as antioxidants against hydrogen peroxide induced oxidative injury. Food Res. Int..

[51] Lapsongphon N., Yongsawatdigul J. (2013). Production and purification of antioxidant peptides from a mungbean meal hydrolysate by *Virgibacillus* sp. SK37 proteinase. Food Chem..

[52] Tang C.H., Wang X.S., Yang X.Q. (2009). Enzymatic hydrolysis of hemp (*Cannabis sativa* L.) protein isolate by various proteases and antioxidant properties of the resulting hydrolysates. Food Chem..

[53] Zou T.B., He T.P., Li H.B., Tang H.W., Xia E.Q. (2016). The structure-activity relationship of the antioxidant peptides from natural proteins. Molecules.

[54] Rajapakse N., Mendis E., Jung W.K., Je J.Y., Kim S.K. (2005). Purification of a radical scavenging peptide from fermented mussel sauce and its antioxidant properties. Food Res. Int..

[55] Saiga A., Tanabe S., Nishimura T. (2003). Antioxidant activity of peptides obtained from porcine myofibrillar proteins by protease treatment. J. Agric. Food Chem..

[56] Halliwell B. (1988). Albumin-an important extracellular antioxidant?. Biochem. Pharmacol..

[57] Roche M., Rondeau P., Singh N.R., Tarnus E., Bourdon E. (2008). The antioxidant properties of serum albumin. Febs Lett..

